# Status of stigma on the health care workers related to COVID-19 at the first wave of the pandemic in Iran: A qualitative study

**DOI:** 10.3389/fpsyt.2022.1045683

**Published:** 2022-11-03

**Authors:** Rahim Badrfam, Mostafa Qorbani, Atefeh Zandifar

**Affiliations:** ^1^Department of Psychiatry, Roozbeh Hospital, Tehran University of Medical Sciences, Tehran, Iran; ^2^Non-Communicable Diseases Research Center, Alborz University of Medical Sciences, Karaj, Iran; ^3^Chronic Diseases Research Center, Endocrinology and Metabolism Research Institute, Tehran University of Medical Sciences, Tehran, Iran; ^4^Social Determinants of Health Research Center, Alborz University of Medical Sciences, Karaj, Iran; ^5^Department of Psychiatry, Imam Hossein Hospital, School of Medicine, Alborz University of Medical Sciences, Karaj, Iran

**Keywords:** stigma, health care workers, COVID-19, perplexity, mental health

## Abstract

**Background:**

Stigma can be seen as a mark of disgrace that can lead to the separation of one person from another. In this qualitative study, we assess the status of stigma among in front-line health care workers (HCWs) during the first wave of the COVID-19 pandemic in Iran.

**Subjects and methods:**

The participants were selected from frontline HCWs related to COVID-19 in Imam Ali and Imam Hossein referral hospitals in Alborz province, Iran. Study was conducted between May and June 2020. The 32-item checklist Consolidated Criteria for Reporting Qualitative Research (COREQ) was used to report this qualitative study. Interview questions were prepared based on the grounded theory method. The thematic approach was used to analyze the data content. Data analysis was based on open and axial coding and after implementing the codes in MAXQDA software.

**Results:**

The results of this study included 4 themes, 8 categories and 33 sub-categories. Themes included extrinsic and intrinsic elements of stigma, perplexity and stigma removal requirements. Extrinsic elements included “creating blame and shame” and “discrimination” categories. Intrinsic elements included “the desire to be avoidance,” “feeling depressed and frustrated” and “feeling anxious and scared” categories. Perplexity included “feeling loss” category. Stigma removal requirements included “factors causing stigma” and “protective agents against stigma” categories.

**Conclusion:**

Low public awareness on COVID-19 and inadequate public care, limited personal protective equipment and inadequate facilities for HCWs along with lack of appreciation for their efforts, lack of proper psychiatric/psychological counseling to identify and treat symptoms associated with mental health and the limitations of training to maintain mental health skills are considered to be factors in the formation of stigma among HCWs related to COVID-19. Health policymakers should implement coherent strategies related to increasing public awareness and providing personal protection needs and counseling care for HCWs in relation to COVID-19.

## Introduction

Stigma can be seen as a mark of disgrace that can lead to the separation of one person from another (1) and can be evaluated in different layers. Personal stigma can be considered as negative attitudes toward others; just as perceived stigma can be considered as perceived attitudes of others and self-image can be considered as a self-attribution of negative attitudes of others (2).

According to some studies, mental illness stigma has a negative effect on many life domains including housing (3), employment (4), income (5, 6), public perceptions about resource allocation (7) and access to treatment and care (8, 9). Such results underscore the economic usefulness of stigma-reduction interventions (10, 11).

With the emphasis of social aspect, stigma is defined as a process that is used to exclude those who are known as a potential source of disease and may be considered a threat to the effective social living in the society (12). In the meantime, other views on the effect of stigma on the individual, community and state levels in the formation of social inequality have been proposed (13). Experts pay attention to these inequalities in mental health issues, emphasizing the cultural aspect. These cultural issues in this area include concepts such as access and quality of receiving services, family experiences (such as secrecy for the sake of family), specific cultural beliefs and negative emotional responses (self-stigma) (14).

In the health system and in the widespread recent pandemic conditions, especially in case of prolongation of the pandemic time or even after its end, stigma can cause major challenges in the health economy due to the destroying the image of the safety of reference hospitals and the possible decreasing in the number of referrals to these medical centers (15). On the other hand, in addition to the economic aspects of stigma, which are of great attention, other social, psychological, and cultural effects are also noteworthy (10).

In some opinion, stigma related to novel coronavirus disease (COVID-19), like other aspects of the disease, can have a different meaning and can go beyond individual sense (16). This is especially important when it comes to healthcare staff, because of their impact on the care of patients with COVID-19 and pandemic control (17). Both stress in and out of the workplace can play a role in the formation of anxiety among health care workers (HCWs) (18). For this reason, especially in relation to COVID-19, it is important to pay attention to the mental health status of HCWs and its influencing factors (19, 20).

In COVID-19, stigma can mean discrimination against a group of people (for example HCWs, patients, or their families) that can be related to factors such as lack of knowledge about the disease and its spread, along with fear and anxiety related to it (16). Bhanot et al. mentions an example of these cases including people’s stigmatizing reactions toward deceased relatives in the context of COVID-19 due to strong irrational fear and threat associated with the virus. They have also mentioned the stigma toward the forefront HCWs in India during the peak of the pandemic by neighbors, landlords, taxi drivers and even family members and have emphasized the equality of all human beings based on nature and avoiding division (12).

Stigmatization of other infectious diseases in the past, such as sever acute respiratory syndrome (SARS), has been prominent in both patients and HCWs. Fear of being stigmatized and of being quarantine-related deprived of various social positions and opportunities has also been seen among this group of people (21). These points, along with other problems with COVID-19-related economic issues, can exacerbate existing problems (22).

The uncertainty associated with COVID-19, due to the unpredictability of various conditions and changes related to infection control methods and public health recommendations, can provide the background for stigma among HCWs (21, 23). On the other hand, caring for patients at high risk of serious infections is associated with significant stigmatization. Meanwhile, it appears to be associated with long-term risks for HCWs, even after the end of quarantine and pandemic conditions (24). Thus, it is important to pay attention to the underlying factors that cause stigma among HCWs during the COVID-19 pandemic.

With various studies on HCWs associated with COVID-19 showing a relatively high prevalence of anxiety and depression symptoms among HCWs, stigma seems to be a worrying trend for this group of workers, especially frontline workers (25–27). In a qualitative study in Nigeria, Kwaghe et al. investigated stigma and traumatic experiences among 19 frontline healthcare workers during the COVID-19 pandemic. They have mentioned themes such as “Early stage of the pandemic” including fear, anxiety and socio-economic effects of the pandemic, “working with COVID-19 patients” including duty stress, “psychological trauma” and “stigmatization” from colleagues, family and friends and its reasons including fear of infection, limited knowledge of the virus and working in the isolated environment as the results of their qualitative study. They have mentioned stigmatization as a big challenge for frontline HCWs in performing their duties. They have also emphasized the psychological impact of stigma experienced by them in reducing the quality of services provided to patients (28). Ramacy et al., in a study involving 260 HCWs in a hospital in southern Italy, examined Social Stigma during COVID-19 and its impact on HCWs Outcomes. They reported stigma as a positive predictor of burnout and a negative predictor of satisfaction (24). In a study in Nepal, Adhikari et al. investigated the prevalence of anxiety, depression, and perceived stigma among HCWs related to COVID-19 and reported the prevalence of anxiety and depression among 213 HCWs, 46.95 and 41.31%, respectively. They mentioned the experience of some form of perceived stigmatization due to COVID-19 among HCWs, 57% and mentioned that frontline HCWs were 6 times more stigmatized than non-front line HCWs (29).

Stigma related to some infectious diseases such as HIV is common among HCWs in Iran (30). After the COVID-19 pandemic, the issue of stigma among HCWs received special attention. In a study in Iran during the peak of the COVID-19 pandemic, which examined posttraumatic stress symptoms and stigma among 894 front-line HCWs in 9 general hospitals, a strong and positive significant correlation was reported between their scores (coefficient: 0.83) (31).

During the outbreak of COVID-19, Iran was affected by this disease early on, and despite the use of existing facilities and the acceptable capacity of primary health care, it faced large waves of outbreaks (32). Meanwhile, HCWs, especially frontline workers, are exposed to mental health problems due to exposure to high workload and caring for patients with serious and highly communicable diseases with the need for long-term hospitalization (33, 34). Azizi et al. reported the prevalence of the physical and psychological anxiety among HCWS 47.9 and 70.5%, respectively. They also reported the prevalence of depression, anxiety, and stress Symptoms among them 44.8, 43, and 34.8%, respectively (35). Rayani et al. also reported significant negative relationship between anxiety and resilience in a study of 550 HCWs related to COVID-19 in Iran (36).

In this way, considering the high prevalence of symptoms of mental health disorders among HCWs in Iran during the COVID-19 pandemic, we hypothesize that in-front line HCWs related to COVID-19 are suffering stigma related to their employment status in the family, occupational, and community environments. Here, based on grounded theory, we discuss the interaction of HCWs with their inner understanding of themselves and its interaction with the surrounding environment, including a set of contacts from close family layers to community levels. Such an approach in this field, in our hypothesis, becomes the basis for the formation of perceived stigma and self-attribution of negative attitudes of others in HCWs during the COVID-19 pandemic. Such experience, along with everyday events and current limitations, presents mental health disorders as a serious challenge among HCWs. In the following, based on the experience gained from these concepts, we will express the solutions to deal with them among this group of people in the society. According to this, in this qualitative study, we assess the status of stigma among “in front line” HCWs during the earliest wave of COVID-19 outbreaks in Iran.

## Materials and methods

### Participants

Participants were selected using convenience sampling method among the personnel involved in the care of COVID-19 patients who worked in various internal medicine and infectious wards and intensive care units (ICU) of the two large general hospitals of Alborz province at the first wave of the COVID-19 pandemic in Iran. They included physicians, nurses, and paramedics. They were invited to participate in an individual interview by sending a text message to them via mobile phone. In addition to requesting a response to agree or disagree to participate in the study by replying to the sent message, one of the interviewers, in accordance with personal protection standards, followed up the receipt of the messages by the participants and their response to voluntarily attend the interview.

The inclusion criteria for the study included all HCWs working in two hospitals of the study site who were older than 18 years and were physicians, nurses and paramedics involved in the care of COVID-19 patients who were working in different wards of the referral hospital for the care of patients with COVID-19. These individuals were HCWs who, after stating the objectives of the study, tended to describe their situations to others. Sampling and coding continued until no new code was obtained and saturation occurred. Thus, on this basis, sampling was completed and the sample size was determined. Exclusion criteria included people who had a previous history of psychiatric disorders under drug treatment in the last year (in the form of self-report).

Finally, the number of participants in this study was 20 [14 females (70%) and 6 males (30%)]. They included 5 physicians (medical doctor), 12 nurses (bachelor’s and postgraduate degree) and 3 paramedics (post-diploma degree). Paramedics included radiology and laboratory technicians, social workers, and the like. All these occupational groups had close and direct contact with patients infected with COVID-19 in different departments of the hospital during their stay in the hospital. The mean age of the participants was 33.1 (*SD* = 7.22) years with range of 21–56 years old. Five participants were in the ICU, five in the infectious diseases ward and ten in the internal medicine ward. The average years of work experience of HCWs was 8.1 (*SD* = 6.30) years, which included at least 1 year and a maximum of 22 years (Table 1).

**TABLE 1:** Demographic characteristics of study participants.

		Number (percentage)/average (Standard Deviation-SD)
Gender	Female	14 (70)
	Male	6 (30)
	Total	20 (100)
Job	Physician	5 (25)
	Nurse	12 (60)
	Paramedic	3 (15)
Mean age (years)		33.1 (SD = 7.22)
The average years of work experience (years)		8.1 (SD = 6.30)

All participants and interviewers were Iranian and the interview was conducted in Persian. This study was conducted between May and June 2020.

### Questions and description

The 32-item checklist Consolidated criteria for reporting qualitative research (COREQ) is used to report this qualitative study (37).

Questions asked of HCWs during the interview included the following:

1) Have you been blamed and embarrassed by the working conditions of the Coronavirus pandemic? How did you experience this blame and shame?
2) What are the sources of this blame and shame in the work environment caused by the Coronavirus pandemic?
3) Have you experienced a sense of rejection in the workplace or in everyday life? What were the sources?
4) Have you experienced an inability to understand the environment? Can you explain this feeling?
5) Explain your sources of information about the pandemic. What problems do you feel in the context?
6) During the pandemic period so far, how has your personal and professional life changed?
   How has the recent pandemic affected your personal and professional life?
7) What do you think about your protective equipment in the workplace and your expectations of officials?

### Research team and reflexivity

#### Personal characteristics

The interview was performed by two male and female expert psychiatrists in qualitative research. At the time of the interview, the two interviewing psychiatrists were in charge of the mental health team at a mental health counseling clinic.

#### Relationship with participants

Prior to the interviews, the interviewees spoke to HCWs, observing health protocols and using personal protective equipment. This initial talk was conducted for a short period of up to 15 min due to the pandemic conditions. The session was performed in a well-ventilated room outside the ward of patients with COVID-19. Only one interviewer and one HCW were present in the room during each initial interview. These conditions were met for all initial interviews in the same format and under the same conditions. In order to facilitate the conditions of the main interview, a proper rapport was formed with the HCWs in these meetings. This was done in the form of holding a meeting with a friendly approach and in order to create a sense of confidence in trying to improve the personal and working conditions of HCWs.

In this short communication, which was done in order to get acquainted with the people who met the inclusion criteria, the objectives of the study and the reasons for doing so were discussed.

### Study design

#### Theoretical framework

The grounded theory method was used to underpin the study (38). Grounded theory is a known methodology in research studies. This method deals with the discovery or creation of theory from data that has been systematically obtained and subjected to comparative analysis (39). The focus here is on discovering patterns of social life that people may or may not be aware of (40). In fact, a diverse approach is considered here, which studies a kind of interaction between the individual and the surrounding environment (41). Grounded theory, especially in triangulation with hermeneutics, is a way to understand complex human phenomena that explains basic social processes at a higher level of abstraction. This path is intended as a framework for planning quantitative interventions (42). In this way, grounded theory and thematic analysis can prevent the bias of the results by using mixed methods and multiple sources and coders and increase the validity of the findings (43).

A grounded theory is produced based on themes. Themes are also formed based on the data and analysis process. In this way, they reveal experiences and achievements from different situations and fields. In this way, it reveals the experiences and understanding of meaning from different situations and contexts (44).

#### Participant selection

Sampling was based on convenience and consecutive method (45). The interview was conducted as a face-to-face meeting with appropriate physical distance and in an environment outside emergency department and patients’ wards and in a relaxed manner, in the form of a session of about 30 min and at the time intended by the participants. The conditions of the interviews were similar to the initial interviews with the HCWs. The total number of participants in the study was 20, including 5 physicians, 12 nurses and 3 paramedics. While identifying cases to enter the study, two HCWs who met the study criteria, stating that they did not have enough time to participate in the study, but after selecting the final 20, all participated in the study.

#### Setting

The study was conducted in Imam Ali and Imam Hossein hospital centers of Alborz province, Iran. These two hospitals are general academic hospitals belonging to Alborz University of Medical Sciences. At the time of the study, a number of internal, infectious and ICU wards in these hospitals were dedicated to the care of patients with COVID-19. Imam Ali and Imam Hossein hospitals have 498 and 150 active inpatient beds, respectively and at the time of the study, 140 and 48 inpatient beds were assigned to patients with COVID-19, respectively. In Imam Ali Hospital, 110 and 30 beds were reserved for isolated COVID-19 wards and ICU for COVID-19 patients, respectively. This number included 36 inpatient beds and 12 ICU beds in Imam Hossein Hospital.

The interview took place in a specific room on one of the floors of each hospital clinic, which was set aside separately during the interview. At the time of the interview, only the interviewer and the HCW were present at the interview site. The interviews were conducted during the first wave of COVID-19 pandemic in Iran, with the aim of familiarizing with the state of stigma among HCWs and the relevant assessment needed to improve the situation.

#### Data collection

The questions, questionnaires and guidelines were designed and specified before the study.

Prior to the start of the study, each interviewee conducted an interview with another member of the group who had the inclusion criteria to enter the study but was not a participant in the study. All interviews were conducted in one session and were not repeated.

The work process was such that first and after the initial conceptualization, the desired references were searched in the literature. Previous studies in this field, especially regarding the status of stigma during past pandemics and other related concepts, were evaluated and the results of those studies and their methodology were discussed (46–49). Next, related primary questions were designed and edited several times based on related studies up to that time. The initial pilot study was conducted in the form of three initial interview sessions and recorded for further evaluation. After the end of each session, a discussion was held about the content of the interview with the aim of obtaining the best and most accurate questions related to the interview with HCWs. The holding of these three pilot sessions was accompanied by some changes in the questions and the method of conducting the interview, including trying to make the interviewee-centered sessions.

With the written consent of the participants before starting the interview session, the conversations in the interview session were recorded as audio recording. Each session lasted about 30 min.

### Analysis and findings

#### Data analysis

Out of a total of 20 interviews, 142 codes were obtained. These codes were eventually placed in 4 them and 8 categories, which included a total of 33 sub-categories. Based on grounded theory (38), the development of a coding tree was as follows:

With the help of the deductive process, based on the conceptual framework of study and its objectives, first the identification of general data categories was done, and then a correct understanding of the themes and initial data was obtained. Themes were obtained based on the data. Based on these themes and related details, more specific coding categories have been obtained (50). Data analysis was based on open and axial coding and after implementing the codes in MAXQDA software. After presenting the results to the participants, at the appointed time, some of them provided feedback over the phone, and their comments were applied in subsequent settings.

#### Reporting

In this report, participant quotations were presented to reveal the themes and findings of the study. Participant quotations were presented in the form of a table according to the themes, categories and subcategories obtained. There was consistency between the data presented and the finding. The main themes were clearly defined. The report also discussed various findings and sub-themes.

## Results

Four themes were extracted in this study. Eight categories and 33 sub-categories were other components of results of the study (Table 2).

**TABLE 2:** Themes and their categories related stigma over health care workers related to COVID-19.

Theme	Category	Subcategory	Participant explanatory quotations
**Extrinsic elements of stigma**	**Creating blame and shame**	**Blaming look at the staff**	*My daughter has allergies. She has been coughing for a few days and her eyes were red. During this time, my wife has repeatedly argued with me that it is your fault.* “*If something bad happens to my child, I will not forgive you.” While my daughter has a recurrence of allergies every spring.*
		**Misjudgment and labeling**	*They think that because we are the medical staff, we will definitely get COVID-19*; “*Oh my God, let your family come to us,” said my mother-in-law several times. You deal with the patient every day. Even now, I have heard that you have shortness of breath. You make children sick*
		**Curiosity about getting sick**	*My mother calls every day and asks, “Didn’t you and your husband and children get Corona?” It’s as if they’re waiting for us to get Corona.*
		**Avoidance behaviors**	*Because they know I’m a medical staff, they don’t even let their kids play with my kids; My mother-in-law repeatedly told me directly and indirectly, “Don’t come to our house.*
	**Discrimination**	**Repulsive and cautious behaviors of those around and society**	*I went to the medical goods store to buy. When I told the salesman that I wanted shield and glasses for myself for hospital, he shouted at me and said, “Madam, go back, your bag is hitting the table, now we’re all taking Corona.”; In the parking lot, the neighbors, who know that I work in the hospital, as soon as they see me, quickly turn their backs on me and run away from me.*
**Intrinsic elements of stigma**	**Feeling rejected and the desire to be avoidance**	**Refuse to take leave**	*I can’t wait to see anyone. I feel like everyone is looking at me the same way.*
		***Hide possible symptoms of the disease***	For *a day or two, I felt dizzy and sometimes itchy in my throat. I really doubted I was sick. I didn’t want to talk to anyone, not even my wife. I was both afraid to worry and pessimistic about myself and my job.*
		***Feeling lonely***	*My wife and child are reluctant to see me every time I return home. It’s as if they’re afraid to see me. I really feel lonely and confused. It feels so bad to feel like I have to be in quarantine for days.*
		***Fear of being fired or unemployed***	*Honestly, I’m contracted, and if I make a mistake or miss a few days, I’ll lose my job.*
	**Feeling depressed and frustrated**	**Disappointment with the future**	*No one understands us properly, and soon everyone will forget how much we suffered.*
		***Sadness***	*As before, I can’t be happy. I feel sad all the time.*
		***Decreased self-confidence***	*We are told that you are so exposed and infected that we no longer feel that self-confidence.*
		**Suicide thoughts**	*A few days ago, three patients died in my shift. I felt weak. The loneliness and confusion of those around me was further compounded by the thought of suicide. But I did nothing.*
	**Feeling anxious and scared**	**Anxiety caused by sleep disturbances**	*The possibility of rest is low. My sleep conditions are disturbed. I am all asleep and awake. I have nightmares at night. It’s as if something very bad has happened.*
		**Feeling tired and exhausted**	*Deaths are also high. These make a person tired and exhausted.; Sometimes I wish I had another job not to deal with the patient.*
		***Guilt feeling***	*When my child coughs, I feel guilty. My conscience is really bothering me that I have this job.*
		***High working pressure***	*The number of clients is very high and it is difficult to control them and it leads to anxiety in us*.
		***Physical symptoms and anxiety***	*I’ve already had dry coughs before. Now it’s more, but I have no other symptoms.*
**Perplexity**	**Feeling loss**	**Detachment**	*Sometimes when people around me treat me unnaturally, I feel like I’m confused;* *I don’t know how to explain that feeling. I feel like I’ve loss something.*
		***Melancholia***	*Sometimes I feel like I’ve lost my inner capital; it’s a very painful feeling. It’s a feeling of emptiness.*
**Stigma elimination requirements**	**Factors causing stigma**	**The role of the media**	*TV programs are not complete. What they show is a form of news that often exacerbates stress and takes society’s view of medical staff toward those as high risk (for transmitting the infection).*
		**The role of cyberspace**	*The information is still incomplete.* *The role of the Internet is very important. Rumors and stigma may spread through both people and cyberspace.*
		**Lack of knowledge about the disease**	*The disease is still very unknown and even we do not know much about it. I think this is very worrying and misleading.*
		**The role of insufficient information of the people**	*People around us, unwittingly and without accurate information, always think that we must be sick, or that we are sick, or that we are carriers of the disease.*
		**Living and economic issues**	*In society, economic problems abound. People are much more concerned about their own health, especially given the needs of the family. Therefore, those who feel they may be ill may be treated badly.*
	**Protective agents against stigma**	**Sufficient equipment**	*Occupational safety and standards are low. Maybe if our safety and equipment is perfectly adequate, people around us will know that we don’t have to be sick. The same is true for ourselves.*
		**Adjust hospital attendance**	*Our shifts are too much. The number of staff is small and most of us are on duty one day in between.*
		**Improving the level of care**	*Some are clearly negligent and put themselves and others at risk. Training is very important in this regard.*
		**The presence of psychologists and psychiatrists alongside staff**	*Our job is always stressful. We are always subject to judgment. The situation is much worse now. Psychologists and psychiatrists should really be with us.*
		**Ensuring job security**	*I am a company employee and I am not an official employee and I have not been paid for months.*
		**Pay attention to the positive aspects of the job**	*It feels good to serve patients.*
		**Appreciation of the treatment staff**	*We like to be respected. Finally, one day Corona will be controlled. We must not forget.*

Themes include “extrinsic elements of stigma,” “intrinsic elements of stigma,” “perplexity in the ground of stigma” and “stigma removal requirements.” Each category has a number of sub-categories.

### Extrinsic elements of stigma

Here we are faced with the concept of being blamed for being in a care setting related to COVID-19 by those close to the HCWs. Also, the wrong beliefs about being infected with infectious disease by taking care of these patients are mentioned by HCWs. Being discriminated against due to job status by sellers and other job groups at the community level is another point worth mentioning.

This theme has two categories. The categories include “creating blame and shame” and “discrimination.”

**(1) Creating blame and shame** include the following sub-categories:

**Blaming look at the staff** (Female, nurse, 31 years old: “*My daughter has allergies. She has been coughing for a few days and her eyes were red. During this time, my wife has repeatedly argued with me that it is your fault.”)*,**Misjudgment and labeling** (Female, nurse, 31 years old: They think that because we are the medical staff, we will definitely get COVID-19; “*Oh my God! let your family come to us*,” said my mother-in-law several times.),

**Curiosity about getting sick** (Female, nurse, 30 years old: “*My mother calls every day and asks: “Didn’t you and your husband and children get Corona?” It’s as if they’re waiting for us to get Corona.”*),** Avoidance behaviors** (Female, physician, 35 years old: “*Because they know I’m a medical staff, they don’t even let their kids play with my kids”).*

**(2) Discrimination** includes the following sub-category:

**Repulsive and cautious behaviors of those around and society** (Female, nurse, 31 years old*: “I went to the medical goods store to buy. When I told the salesman that I wanted shield and glasses for myself for hospital, he shouted at me and said, “Madam, go back, your bag is hitting the table, now we’re all taking Corona”).*

#### Intrinsic elements of stigma

Here we are associated with the concept of worries and fears due to the possibility of being infected with the disease and interpreting the usual symptoms as symptoms of COVID-19. The worry of job loss due to possible infection is one of the other points mentioned in this field. The internal interpretation of the evasive behavior of the family and attributing it to the possibility of being infected due to special working conditions, decreased self-confidence and feelings of sadness and loneliness are mentioned by the HCWs. Also, caregivers report anxiety, excessive worry, and poor sleep due to frequent exposure to a large number of clients and the inappropriate vital condition of patients and their death in inpatient wards.

This theme has three categories. The categories include the desire to be avoidance, feeling depressed and frustrated and feeling anxious and scared.

**(1) Feeling rejected and the desire to be avoidance** includes the following sub-categories:

**Refuse to take leave** (Male, paramedic, 22 years old, *“I can’t wait to see anyone. I feel like everyone is looking at me the same way.”)*, ***hide possible symptoms of the disease*** (Female, nurse, 42 years old, *“For a day or two, I felt dizzy and sometimes itchy in my throat. I was both afraid to worry and pessimistic about myself and my job.”)*, ***feeling lonely*** (Male, physician, 39 years old, *“My wife and child are reluctant to see me every time I return home. It’s as if they’re afraid to see me”)*, ***fear of being fired or unemployed*** (Male, nurse, 25 years old, *“Honestly, I’m contracted, and if I make a mistake or miss a few days, I’ll lose my job”).*

***(2) Feeling depressed and frustrated*** includes the following sub-categories:

**Disappointment with the future** (Female, nurse, 40 years old, *“No one understands us properly, and soon everyone will forget how much we suffered.”)*, ***sadness*** (Female, nurse, 31 years old, *“As before, I can’t be happy. I feel sad all the time.”)*, **decreased self-confidence** (Male, nurse, 31 years old, *“We are told that you are so exposed and infected that we no longer feel that self-confidence.”)*, **suicide thoughts** (Female, physician, 31 years old, *“A few days ago, three patients died in my shift. The loneliness and confusion of those around me was further compounded by the thought of suicide”).*

**(3) Feeling anxious and scared** includes the following sub-categories:

**Anxiety caused by sleep disturbances** (Female, paramedic, 31 years old, *“The possibility of rest is low. My sleep conditions are disturbed. I am all asleep and awake”)*, **Feeling tired and exhausted** (Male, physician, 32 years old, *“Deaths are also high. These make a person tired and exhausted”*), ***Guilt feeling*** (Female, nurse, 31 years old, *“When my child coughs, I feel guilty.”)*, ***High working pressure*** (Female, nurse, 21 years old, *“The number of clients is very high and it is difficult to control them and it leads to anxiety in us”)*, ***Physical symptoms and anxiety*** (Female, nurse, 31 years old, *“I’ve already had dry coughs before. Now it’s more, but I have no other symptoms”)*.

#### Perplexity

Here, we are faced with concepts such as a feeling of separation from the environment, confusion and loss among HCWs when faced with the unusual behavior of others and changing environmental conditions.

This theme has one category. This category includes “feeling loss.”

**(1) Feeling loss** include the following sub-categories:

**Detachment** (Female, nurse, 31 years old, *“Sometimes when people around me treat me unnaturally, I feel like I’m confused; I don’t know how to explain that feeling.”*), **Melancholia** (Female, nurse, 26 years old, *“Sometimes I feel like I’ve lost my inner capital; it’s a very painful feeling”).*

#### Stigma elimination requirements

Here, the causal and protective factors of stigma in HCWs’ conversations are considered. They describe the problem of spreading rumors and wrong news in the virtual space and among people on the one hand, and the unknown aspects of the disease on the other hand, as worrying. They emphasize the necessity of having appropriate and complete personal protective equipment and mention that having such protection can reduce people’s feeling that they are sick and people’s negative attitudes toward them. Also, they emphasize the need to manage their working hours by the hospital officials and reduce related pressures in this field. Emphasizing on stressful working conditions, they point out the need to be supported by psychological and psychiatric counseling systems. They also emphasize the need to extend emotional care and support to them in order to maintain their morale and strengthen their hope and steadfastness in hard working conditions.

This theme has two categories. The categories include “factors causing stigma” and “protective agents against stigma.”

**(1) Factors causing stigma** include the following sub-categories:

**The role of the media** (Male, nurse, 56 years old, *“What TV programs show is a form of news that often exacerbates stress and takes society’s view of medical staff toward those as high risk for transmitting the infection*.”), **The role of cyberspace** (Female, nurse, 42 years old, “*The information is still incomplete. Rumors may spread through both people and cyberspace*.”), **lack of knowledge about the disease** (Female, physician, 35 years old, *“The disease is still very unknown and even we do not know much about it. I think this is very worrying”)*, **The role of insufficient information of the people** (Female, nurse, 31 years old, *“People around us, unwittingly and without accurate information, always think that we must be sick”)*, **Living and economic issues** (Male, nurse, 56 years old, “*In society, economic problems abound. People are much more concerned about their own health*”).

**(2) Protective agents against stigma** include the following sub-categories:

**Sufficient equipment** (Female, nurse, 35 years old,” *Maybe if our safety and equipment is perfectly adequate, people around us will know that we don’t have to be sick”)*, **Adjust hospital attendance** (Female, paramedic, 29 years old, *“Our shifts are too much. The number of staff is small and most of us are on duty one day in between.”)*, **Improving the level of care** (Female, physician, 31 years old, *“Some are clearly negligent and put themselves and others at risk. Training is very important in this regard.”)*, **The presence of psychologists and psychiatrists alongside staff** (Female, physician, 35 years old, *“Our job is always stressful. The situation is much worse now. Psychologists and psychiatrists should really be with us*.”), **Ensuring job security** (Female, nurse, 26 years old,” *I am a company employee and I am not an official employee. I have not been paid for months.”)*, **Pay attention to the positive aspects of the job** (Female, nurse, 30 years old, *“It feels good to serve patients.”*), **Appreciation of the treatment staff** (Female, nurse, 40 years old, *“We like to be respected. We must not forget”).*

## Discussion

Being blamed by family and friends in family, professional and social environments, due to their job position as HCWs, is one of the salient points taken from the content of interviews with HCWs in our study. Attributing the non-specific symptoms of this group of staff to the symptoms of the COVID-19 and continuing to ask questions about their possible infection, along with creating some emotional and social deprivations for the HCWs and their families, are the bases for the formation of shame in them. This feeling of shame can induce negative experiences with worthlessness and inferiority that along with guilt, can create and intensify stigma among HCWs (51, 52).

In our study, HCWs also expressed concern about discrimination against them and their families in and out of hospital settings. This sometimes led to attempts to hide some suspicious symptoms of COVID-19, which was due to concerns about losing job opportunities in the face of possible discrimination. Also, the precautionary and repulsive behaviors of those who found out about the job status of the staff were also noteworthy.

This is a major threat to HCWs, which not only expose them to bullying and harassment, but can also be associated with an increased risk of perceived stigma (53). This point in relation to social, psychological and medical variables, along with the loss of respect in society, provides more background for stigma and emotional and physical violence against HCWs and can have many long-term consequences in relation to these variables (54, 55). It seems that attention to all the above variables by health policy makers at different individual and social levels is an undeniable part of COVID-19 pandemic management.

Such variables, both at the level of nations and at the level of governments, emphasize the need to evaluate and determine care strategies. In this regard, the role of policy makers in promoting public awareness and the use of public education with effective methods and the role of HCWs as reference groups in forcing policymakers to take responsibility and creating appropriate physical conditions and financial support, is undeniable (56, 57).

The participants in our study talked about the feeling of loneliness, rejection, feeling of some kind of emotional separation, anxiety about the job situation and fear of being fired from work. These had caused the feeling of rejection and the tendency to avoid in them and finally led to the formation of stigma both in the work and family environment. In a similar study in Italy during the COVID-19 pandemic, a group of nurses experienced stigma in their work environment and stigma in everyday lives, which caused them to avoid being close to others (58).

The content mentioned by the HCWs in our study includes components such as blaming, misjudgment and labeling, curiosity and repulsive behavior. These are associated with a number of intrinsic elements, along with psychological distress, and in some cases with psychiatric symptoms such as anxiety and depression and physical health problems (59). According to some studies, these are conditions that can be more severe in a situation that HCWs have a history of COVID-19 (60). Following the SARS epidemic, Ho et al. examined the status of staff between the two groups with and without a history of the epidemic disease. They cited the fear of infecting their families as one of the common features of the two groups and noted concerns about discrimination among HCWs with a history of SARS and fears of contracting the disease among the second group (61).

Also, during SARS epidemics, the rate of stigmatization, such as psychologic distress, was higher among people who were in the front line (62). In fact, among this group of HCWs, there was not only physical fear of illness, but also anxiety caused by stigma and fear of losing patients, and even the behavior of colleagues could be a source of psychological distress.

Verma et al. also noted the association between stigmatization and psychiatric morbidity among HCWs during the SARS epidemic. They cited a decline in performance and an interpersonal relationship as possible consequences of this situation and emphasized the need to address the psychological needs of HCWs and to identify and treat these disorders (49). This is while, in some studies, stigma is seen as a barrier to mental health interventions (63) and health seeking behavior (64). Other examples of this situation include Ramaci et al.’s study of HCWs in COVID-19 in Italy. They described the stigma as a strong predictor for negative outcomes such as fatigue and burnout (24). In our study, HCWs also introduced fatigue and burnout as one of the elements of stigma.

In our study, according to the symptoms of anxiety and depression in the interviewees and their “frontline” conditions, it seems that trying to reduce stigma can be effective in reducing the above symptoms on the one hand, and controlling the symptoms of depression and anxiety on the other hand can play a role in reducing the feeling of stigma. Thus, it seems that trying to raise the awareness of personnel about mental health problems and the need to pay attention to it and perform related diagnostic and therapeutic measures can help improve the above process and reduce the level of stigma among them.

Also, the need to persuade HCWs to express problems related to mental health, such as attention to sleep problems (65) and physical problems related to mental health disorders (66) can be effective in this regard. In our study, part of stigma’s intrinsic elements was anxiety related to sleep disturbances, along with feelings of burnout and anxiety-related physical symptoms. A number of participants also expressed sadness, frustration about the future, and the occasional thought of suicide as part of their mental state.

The participants in our study talk about a sense of loss and perplexity. They mention a feeling like confusion and inability to describe their situation and sometimes they talk about a deep feeling of emptiness. In some similar experiences during the COVID-19 pandemic, it has been reported that the sense of self is affected in terms of detachment and melancholy related to the pandemic. Other similar phenomena have been reported in relation to affecting the sense of self in the context of detachment and melancholy related to the pandemic (67).

According to some experts, this perplexity is a kind of lack of common sense (68). This outward-looking can have similarities with the look “beyond the individual sense” (16). Here perplexity is closely related to the concepts of “detachment” and “melancholia” which were the points we saw in some of the in-front line HCWs who participated in the study.

The impact of media and cyberspace on the intensification of stress related to COVID-19 and their role in the formation of this mindset that HCWs are the source of infectious agent transmission is mentioned by HCWs in our study. In most human cultures, being contaminated and potentially spreading an infectious disease is associated with shame and stigma (69). HCWs in our study also mention incomplete information transmission as one of the salient features of the media during the COVID-19 pandemic. This incomplete information can be associated with many problems related to the performance status of HCWs and cause stigma and discrimination against them in different environments. It can also expose them to the risk of psychological problems (70). A report from Indonesia and Thailand on COVID-19 found that some doctors and nurses were rejected by the community because they were considered a source of virus transmission (71).

In this regard, it seems that in addition to raising awareness at the community level and even in medical service providers to patients with COVID-19, policymakers’ efforts should provide a platform for job stability and job security for HCWs. This is even more important given that these people are at greater risk for COVID-19 and is associated with an increased risk of perceived stigma. As in a study in Iran on a group of HCWs with demographic characteristics and working conditions similar to the HCWs participating in our study, the history of COVID-19 was associated with an obvious increased risk of anxiety, depression, and stress (72). These symptoms are bilaterally related to stigma, especially among HCWs. In fact, in such circumstances, in addition to improving the level of public education, it seems that in the face of the crisis ahead, there is a serious need for synergy between central governments, regional decision makers, community leaders and referral hospital officials (73).

As can be seen, the active elements in stigma appear to be intertwined in many cases, and a combination of intrinsic, extrinsic and some other factors can be effective in its formation in various clinical experiments.

In another study by Maunder et al., on the psychological effects of SARS outbreak, fears of infection and anxiety about transmitting the disease to the family with loneliness were some of the highlights. They reported uncertainty and stigmatization as prominent themes, and reported the presence of anxiety and stress associated with uncertainty among HCWs. Here, the use of psychological and psychiatric counseling in HCW mental health care was emphasized (74). In our study, participants also emphasized the need for psychological and psychiatric services to be available in this situation. This can be helpful in managing the symptoms of anxiety, stress, depression and burn out, as well as helping to reduce stigma.

Another point of the report is the abandonment of work by a number of physician and nurses due to lack of appreciation for sacrificing their lives to help others. In our study, the points made by HCWs as a protective factor against stigma are their emphasis on the need to be appreciated and “seen.” Supporting HCWs and establishing job security seems to be able to play an effective role in reducing stigma among HCWs.

It seems that removing the stigma requires the influence of various factors, and in the social dimensions, creating solidarity and restoring identity according to the experiences that exist in the field of diseases related to stigma can play an important role in this field. Increasing public awareness can also be an effective factor in reducing the stigma associated with COVID-19 (75).

Appropriate interventions related to coping with the current situation and reconstructing the meaning of life using psychological interventions such as positive psychology and other interventions (76) as well as the use of tele-psychiatry capabilities along with local psychosocial support, which can vary from educational planning to emergency interventions (77, 78), can be used and should be considered as a framework for interventions related to the mental health of HCWs.

The usefulness of such interventions may be considered not only from personal and professional aspects related to HCWs, but also by improving patient care, increasing self-confidence and reducing perceived stigma can lead to direct and indirect economic effects while maintaining HCWs efficiency and improving the human resource status of health systems (79).

Given the global burden of COVID-19, it seems that policy implications need to be achieved so that HCWs and health systems can take steps to ensure safety while providing high quality care for patients with COVID-19 (80). Meanwhile, in the field of public health management, it seems that the sustainable exit strategy from the current conditions (81), given the current pandemic situation, needs to have policies that facilitate the personal and professional needs of HCWs. Accordingly, attention to the financial issues of the health care system will be one of the essential issues in major policy-making in the coming years. Thus, as the pandemic continues, clinical and policy strategies to maintain the mental health of HCWs are increasingly needed (82). Creating suitable occupational and environmental conditions accompanied with psychological interventions and paying attention to all groups related to providing services to patients with COVID-19 seems necessary (83).

At the individual and professional levels, some potential measures to reduce mental health problems in HCWs can include effective communication, providing screening facilities and interventions for mental health problems, financial support, actively limiting rumors and misinformation, and legal protections for disability and retirement benefits (84). An important point in this regard can be the correct modeling of other related initiatives in other countries. Establishing evidence-based strategies and close partnership between the government and the community can help in this regard (85).

### Limitations

This study was evaluated based on cross-sectional data, so it cannot be used to investigate the relationship between cause and effect. Due to the limitations related to personal protection measures and the lack of universal vaccination at the beginning of the pandemic, the interviews were held in the shortest possible time with a minimum number of people in each session (one interviewer and one interviewee).

Focusing on the content of the interview and collecting and reviewing the relevant requirements and references before holding the interview sessions and establishing a proper rapport with the participants, a lot of effort was made so that the sessions have high quality and appropriate content despite the limited number and time of the interviews. Also, this study was carried out at the beginning of the spread of the pandemic. The interviews with the participants were accompanied by restrictions due to the observance of the maximum personal protection coverage, and with the proper cooperation and high motivation of the participants in the study, the follow-up of the meeting progressed properly.

## Conclusion

An interview with a group of in-front line HCWs, related to COVID-19, showed that in addition to intrinsic and extrinsic elements, perplexity in the ground of stigma is a major theme in HCWs-related stigma. Feeling loss was the main category associated with this theme, and was associated with concepts related to lack of common sense and beyond the individual sense. Blame and shame were used as extrinsic elements along with feeling rejected, feeling depressed and frustrated, and feeling anxious and scared as intrinsic elements associated with Stigma.

Health policymakers should implement coherent strategies related to increasing public awareness and providing personal protection needs and counseling care for HCWs in relation to COVID-19. Expanding educational programs related to COVID-19 by using reliable scientific sources with more effective participation of experts in this field and raising awareness about the effective role of health workers in the COVID-19 pandemic along with meeting their personal protection needs is an important part in this field.

At the same time, an important part of public education can be done by reference groups of HCWs, during visits or in structured virtual spaces. The role of awareness campaigns in this field, which can be carried out by spontaneous groups of people with the support of health-oriented institutions, can be important and decisive in providing correct information in times of crisis. Also, the expansion of mental health care related to HCWs by considering telepsychiatry for psychological and psychiatric consultations, which is carried out by psychologists and psychiatrists specializing in this field, can play an important role in dealing with stigma among HCWs.

Considering the nature of the pandemic and the sensitivity of its severity to various aggravating factors such as changing strains leading to increased pathogenicity of the virus and protective factors such as universal vaccination, future studies based on the results of this study can include cases that investigate other factors affecting the formation and persistence of stigma among HCWs. Also, paying attention to the persistence of stigma in HCWs and the long-term effects of stigma on the formation of mental health disorders and its consequences can be effective in identifying different dimensions of stigma in the context of the COVID-19 pandemic.

## Data availability statement

The original contributions presented in this study are included in the article/supplementary material, further inquiries can be directed to the corresponding author.

## Ethics statement

The studies involving human participants were reviewed and approved by the Alborz University of Medical Sciences under grant number (IR.ABZUMS.REC.1399.011). The patients/participants provided their written informed consent to participate in this study.

## Author contributions

All authors were equally involved in the study conception and design of the study, searching for articles, and writing the final manuscript, etc.
